# Editorial: A Living History of Immunology

**DOI:** 10.3389/fimmu.2015.00502

**Published:** 2015-09-29

**Authors:** Kendall A. Smith

**Affiliations:** ^1^Division of Immunology, Department of Medicine, Weill Medical College, Cornell University, New York, NY, USA

**Keywords:** immunological history, adaptive immunity history, interleukins, cytokines, lymphokines, T cell antigen receptors

This Research Topic was conceived to provide a venue for investigators to document their critical contributions to our understanding of the cellular and molecular mechanisms that provide our remarkable immune system the capacity to protect us from environmental insults while simultaneously remaining unreactive to our internal molecular milieu. Although the scientific literature gives one a history of what happened, when it happened, and who were responsible, it fails to capture precisely how things came together, and why some investigators were successful, while others working contemporaneously failed. Thus, seminal contributors were asked to recount the various aspects of their experiments and people who were instrumental in making the progress that moved our understanding forward.

Looking back over the brief history of immunology, a discipline that arose after the pioneering approaches of Edward Jenner introduced the smallpox vaccine in 1798 (1) and Louis Pasteur catapulted immunology into universal awareness 100 years later (2–4), the science has only become mature within the past 50 years. For almost 100 years after Pasteur, experimentalists focused on observations of the reactions of whole experimental animals or humans to the administration of putative antigenic substances. For the first time, around 1960, it was appreciated that lymphocytes are the cells that mediate the immune reaction (5–7), and experimentation moved for the first time from *in vivo* to *in vitro*, which allowed one to manipulate and investigate an immune reaction of cell populations “outside of the black box.” During the 1960s, various techniques were improved so that it was possible to discern that several different types of cells cooperated to ultimately generate a measurable immune response, usually monitored by the appearance of antibody-forming cells (AFCs) (8, 9).

As detailed in this Research Topic, by the 1970s, experiments culminated in the demonstration that two distinct types of lymphocytes existed, termed thymic-derived cells (T cells) and bone marrow-derived cells (B cells), which generate AFCs. Furthermore, a third type of cell derived from myeloid cells, termed an antigen-presenting cell (APC), also played a role. Evidence was also presented that there are at least two distinct subsets of T cells. Moreover, investigators detected activities in culture supernatants of activated lymphocyte populations that seemed to enhance or suppress the generation of AFCs as well as the proliferation of various lymphocytes. However, the molecular basis of these activities remained enigmatic and essentially unapproachable, given the experimental biochemical methods then available.

Four new and novel experimental methods were introduced in the 1970s that revolutionized all of biological sciences, which enabled a further reduction from cells to molecules, and led to the discipline that now can be recognized as molecular immunology. In 1972, the flow cytometer introduced a new method to identify and isolate individual cells present in cell populations (10). Also, genetic engineering approaches enabled investigators to identify and isolate complimentary DNA molecules encoding gene products, which then allowed the ready determination of their primary structures, and provided a means to generate essentially unlimited quantities of critical proteins (11). Third, the advent in 1975 of the capacity to isolate and clone individual AFCs, which could produce unlimited quantities of monoclonal antibody molecules (12), could then be used to identify and isolate both individual cells using the flow cytometer, but also new reagents that could be used to isolate and purify individual protein molecules from complex mixtures.

The fourth critical technical advance, which like monoclonal antibodies was also special to immunology, was the creation in 1979 of the methods to select, clone, and grow the functional progeny of individual T cells (13). This advance, for the first time, allowed one to circumvent the tremendous heterogeneity of individual cells within T cell populations so that the molecules responsible for antigen recognition, histocompatibility restriction, and the molecular mechanisms underlying T cell function, including T cell help of antibody production and T cell mediated cytolysis, could be uncovered.

The contributions comprising this compilation of the stories about how the transition from experiments on whole living organisms to cell populations to individual cells and finally to homogeneous molecules represent a unique aspect of scientific communication, in that they tell the behind the scenes dramas that are usually left out of the scientific literature. This Research Topic tells how science gets done, and what the people are like who actually do it. I hope that you enjoy the stories.

## Conflict of Interest Statement

The author declares that the research was conducted in the absence of any commercial or financial relationships that could be construed as a potential conflict of interest.
